# Spindle Cell Carcinoma of the Tongue: A Rare Tumor in an Unusual Location

**DOI:** 10.4061/2011/572381

**Published:** 2011-02-20

**Authors:** Murat Oktay, Tuba Dilay Kokenek-Unal, Bulent Ocal, Guleser Saylam, Mehmet Hakan Korkmaz, Murat Alper

**Affiliations:** ^1^Pathology Department, Diskapi Yildirim Beyazit Training and Research Hospital, 06110 Ankara, Turkey; ^2^Ear-Nose-Throat Department, Diskapi Yildirim Beyazit Training and Research Hospital, 06110 Ankara, Turkey

## Abstract

Spindle cell carcinoma is a rare biphasic tumor consisting of epithelial and mesenchymal components. Presence of this tumor type in the tongue has rarely been reported. Herein, a case of 55-year-old woman who presented with a polypoid lesion at her tongue has been reported. Surgery was performed and pathologic examination revealed a spindle cell carcinoma. We present this rare tumor with an unusual location to contribute in part to the better understanding and awareness of this rare malignancy.

## 1. Introduction

Spindle cell carcinoma (SpCC) is a relatively rare malignancy affecting the upper aerodigestive tract. The most common site of origin in head and neck region is larynx (particularly vocal cords) and hypopharynx. Usually, oral cavity is not affected. SpCC occurs commonly in 6-7th decades of life and represents a male predominance. Smoking, alcohol consumption, and previous irradiation of the head and neck region are the predisposing factors. It is a poorly differentiated variant of squamous cell carcinoma (SCC) [1] with a more aggressive behavior [2–4]. This biphasic tumor is composed of both malignant epithelial and malignant mesenchymal components. Despite several immunohistochemical, electron microscopic, and genetic studies, precise histogenesis of SpCC is quite controversial. This contraversy has reflected itself with a wide spectrum of nomenclature on this tumor type such as carcinosarcoma, pseudosarcoma, sarcomatoid carcinoma, collision tumor, and pseudosarcomatous carcinoma. 

There is limited literature on SpCC arising in the aerodigestive tract. Moreover, there is only a handful of literature reporting the tongue localization of these tumors. We report here a case of SpCC arising from the tongue in a patient who has been previously operated for SCC and treated with radiotherapy (RT).

## 2. Case Report

**Figure 1:**
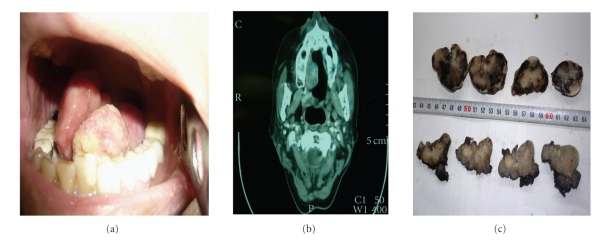
Macroscopic features and radiological imaging of SpCC. (a) Photographic view of the tumor just before operation. (b) Polypoid tumor mass in oral cavity on CT imaging. (c) The cut surfaces of the tumor.

A 55-year-old woman was admitted to the department of otorhinolaryngology with a painless polypoid lesion located in the tongue (Figure 1(a)). Clinical history revealed that she had an unhealing lesion in the right lateral border of the tongue diagnosed with SCC and had been operated twice in 2000 and 2002. She had no metastatic lymph node in performed neck dissection. After the last surgery, she had received postoperative RT. 

On physical examination, a polypoid, exophytic mass was observed in the previously operated region which was 5 × 3.5 cm in size and of firm, rubbery, and nontender character. Laboratory results were within normal limits. Computed tomography scans (CT) revealed an asymmetric, irregular, soft density localized in the right border of tongue (Figure 1(b)). CT and physical examination of the neck region disclosed no abnormality but changes which were compatible with previous operation. Chest X-ray did not show any abnormal findings. Incisional biopsy was performed and showed pleomorphic spindle cells. Together with the previous diagnosis of SCC, the biopsy was diagnosed as SpCC. The patient underwent operation; recurrent polypoid mass was resected with wide surgical margins and additionally bilateral mouth floor resection was performed in combination with left extended supraomohyoid neck dissection. The surgery was finished with reconstruction with myocutaneous pectoral flap. During followup, local recurrence was observed in 3 months, and another operation was performed. Only squamous cell carcinoma was determined in the pathologic examination of the specimen. 

Macroscopically, the bulging mass had been pulled apart and split into two parts by the surgeon. They were 2 × 1.8 × 0.5 cm and 5 × 3.5 × 3 cm in size and had occasional areas of ulceration. The cut surfaces of resected specimen had heterogenous appearance and were firm and whitish in color (Figure 1(c)). Main specimen consisted of 4 incisor teeth, partially mandibula and glossectomy material. In right lateral margin of the tongue, a firm, dirty whitish-colored polypoid tumor with a stalk attaching it to the tongue was observed. It was 3 × 2 cm in size and 1.4 cm in depth on the cut surface.

**Figure 2:**
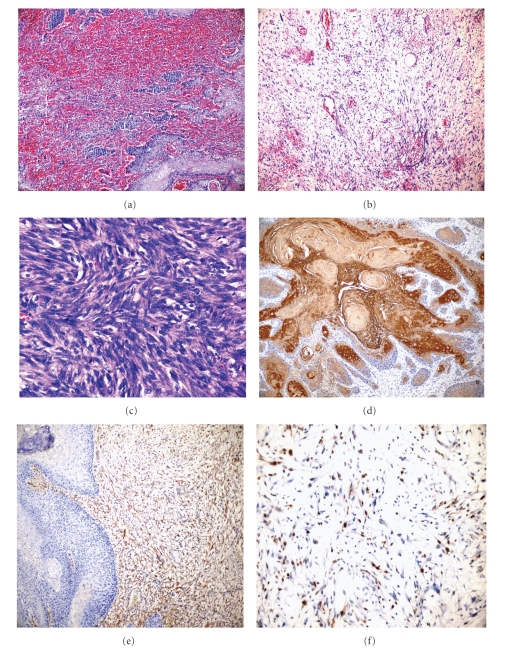
Microscopic features of SpCC. (a) The irregular, atypical spindle cells widely distributed in a loose stroma mimicking granulation tissue. (H&E, ×100). (b) Hypercellular areas of atypical spindle cell component resembling a true fibrosarcoma. Also note those frequent mitotic figures, some of which atypical. (H&E, ×200). (c) “Transition zone of the tumor”, spindle-shaped tumor cells “dropping off” from squamous cell carcinoma. (H&E, ×400). (d) Squamous cell carcinoma component of tumor showing Pan-CK positivity. (Pan CK, ×200). (e) Vimentin positivity in sarcomatoid component. (Vimentin, ×200). (f) Ki-67 staining of stromal spindle cells. (Ki-67, ×400).

Microscopic examination revealed a biphasic tumor composed of keratinized squamous cell carcinoma and spindle cell type sarcomatous stroma. Malign spindle cells, comprising the bulk of the tumor, exhibited hypo- and hypercellular areas and occasional storiform pattern (Figure 2(b)). These cells were pleomorphic with large, oval-round, vesiculated nuclei, and abundant cytoplasm (Figure 2(b)). Bizarre cells, multinucleated giant cells, and frequent mitotic figures, some of which were atypical, were also observed in sarcomatous component (Figure 2(a)). Epithelial component which was moderately to well-differentiated squamous cell carcinoma was easily recognizable throughout the sections but was observed especially in stalk of the tumor. Tumor cells were atypical with increased nuclear/cytoplasmic ratio, having large, pleomorphic, hyperchromatic, vesiculated nuclei, and reasonable eosinophilic cytoplasm. There was no distinct demarcation between two components; a transitional zone could be easily seen in H&E staining (Figure 2(c)). Immunohistochemical markers such as panCK, EMA, and p63 were positive in cells composing the epithelial component and negative in spindle cells of the sarcomatous component (Figure 2(d)). The latter were strongly positive with vimentin whereas negative for SMA, desmin, and HMB-45 (Figure 2(e)). Proliferative index was 60–70% with MIB1 staining (Figure 2(f)). We diagnosed this case as spindle cell carcinoma of the tongue according to the WHO 2005 classification, in which the diagnostic criteria were defined as the presence of malignant spindle cells in addition to the demonstration of invasive or in situ squamous cell carcinoma, or any evidence of epithelial differentiation of spindle cells [5]. Lymphatic, vascular, neural invasion, and lymph node metastasis was not observed but mandibula was invaded by the tumor. The patient was classified as stage IVa according to AJCC guidelines [6] and showed no evidence of disease after a followup of 13 months.

## 3. Discussion

SpCC is a rare variant of SCC which has both malignant squamous cells and malignant spindle cells of epithelial origin. Spindle cell component is responsible for the mesenchymal appearance. And the diagnosis can be challenging, especially when SCC component is not obvious. The spindle cell component may resemble many lesions, ranging from benign reactive ones like radiation-induced granulation tissue to malignant lesions like fibrosarcoma. Thus, to establish a correct diagnosis, any clue of epithelial component should be carefully sought in suspected lesions [5]. Mainly three different theories have been proposed to explain histogenetic nature of spindle cells. First theory is that spindle cells and epithelial cells are arising simultaneously from separate stem cells deserving the name “collision” tumor. Second theory explains the nature of the spindle cell component as an atypical reactive proliferation of the stroma and hence called “pseudosarcoma”. Finally, according to the last theory, cells of both spindle and epithelial components have the same monoclonal origin, and “dedifferentiation” or “transformation” to spindle cells has been occurred [7–9]. Over time, several studies revealed that spindle cells have similar characteristic features with squamous cells in all immunohistochemical, ultrastructural, molecular, and genetic aspects [10]. The presence of a distinct demarcation zone and a different immunohistochemical staining pattern between epithelial and spindle cell components have led some of the researchers to suggest a different origin for these components [11]. However, recently monoclonal hypothesis is widely accepted and is strongly supported by some studies [12–16]. Histochemical and ultrastructural studies showed that spindle cells presented same characteristics with epithelial cells [15]. Additionally, Gupta et al. described that some of the spindle cells or transient cells with mesenchymal appearance express dual antigen-positivity with both epithelial (cytokeratin) and mesenchymal (vimentin) markers [15].

As mentioned above, SpCC, like SCC, affects primarily men in 6-7th decades and is strongly associated with smoking and alcohol consumption [1, 4, 5]. In our case, the age was within typical range but unusually the patient was a woman. Alcohol abuse was not reported but she was a heavy smoker and diagnosed with SCC and received postoperative RT 7 years ago. Exposure to radiation in head and neck region is described as a predisposing factor. SpCC arising from less aggressive types of SCC after RT has rarely been reported [1, 16–19]. Combining the results of five major related studies [17–21], Lewis Jr. reported that 18% of SpCC in aerodigestive tract received previous RT [10]. Regarding specifically the oral region, previous literature indicated the presence of radiation induced SpCC as in our case [22, 23]. Controversial literature also exists [24, 25] with one of them being the most recent with a series of 103 oral SpCC cases and interestingly none of the patients had a prior history of radiation [24]. The author suggested that this may be due to difference in treatment choices between oral and laryngeal cancers as in oral SpCC surgical treatment is preferred instead of adjuvant radiotherapy in contrast to laryngeal cancers [24]. In head and neck region, the most common localizations of SpCC are larynx, oral cavity, skin, tonsil, and pharynx [4, 26]. In our case, the SpCC had arisen from the tongue which is one of the rare sites. Laryngeal tumors clinically present as polypoid exophytic masses and exhibit typical symptoms such as hoarseness, voice changes, airway obstruction, and dysphagia. Oral and oropharyngeal tumors present as a painful or painless masses with nonhealing ulcer, dysphagia, or bleeding [4, 10]. The tumors usually grow up rapidly. Although rapid growth was not the case in our patient, the lesion expressed classical polypoid and occasionally ulcerated appearance. Histologically, mesenchymal component typically forms the bulk of the tumor and epithelial component often blends into it [1]. Malignant pleomorphic cells may exhibit hypo- and hypercellular areas and sometimes storiform pattern. Typical and atypical mitotic figures are usually prominent. Giant cells may also be found. Our case showed the characteristic microscopic features above including the presence of abundant giant cells (Figures 2(a), 2(b), 2(c), 2(d), 2(e), and 2(f)). Although osteosarcomatous, chondrosarcomatous, or rhabdomyosarcomatous differentiation may be present in especially previously irradiated patients, we did not experience such areas [5, 27]. Regional lymph node metastasis is reported up to 25% of cases and distant metastasis is less common [5]. Our patient was free of lymph node metastasis but mandibular bone was invaded. When SpCC metastasizes, metastatic foci may contain SCC alone or both components, and rarely, only spindle cell component [7]. Silvestri et al. have presented that immunologically sarcomatoid elements are always strongly positive for vimentin but cytokeratin reactivity of spindle cells are variable and may be completely absent [7] like it is our case. 

Spindle cell carcinomas should be treated like the squamous cell carcinomas in the same stage. In laryngeal tumors, treatment of choice is primarily surgery with possible neck dissection followed by radiation therapy or chemotherapy [4, 11, 28–30]. However, Thompson et al. reported that surgically treated patients had a better prognosis than the patients treated with radiotherapy alone [17]. In the oral region, surgery is accepted as the best treatment choice. Although in general, there are increasing tendency for conservative approaches in cancer treatment. Controversary on applying RT especially for oral cancers also exists [31]. The role of chemotherapy is not clear yet [3, 11, 32]. Low-stage, small-size, glottic location in larynx, absence of previous radiation, superficial location and polypoid, and exophytic growth are related to better prognosis [8, 28]. In addition, it is indicated that keratin expression of the spindle cells adversely affects the survival [20]. Although SpCC has many similar clinical and histopathological features with SCC, the former is more aggressive and hence has lower overall survival like poorly differentiated SCCs [2, 3, 25]. High recurrence rate of the tumor, even in the early-stage patients, is another important factor affecting survival [3]. In our patient, despite of resection with wide surgical margins, tumor showed recurrence in followup and the patient underwent salvage operation. There is no consensus on the treatment of recurrent tumors. Su et al. reported that there is a statistically significant relationship between overall survival and salvage operation [3, 22]. However, Thompson et al. reported a worse prognosis in patients underwent salvage procedure [17]. 

Differential diagnosis may be problematic especially in cases exhibiting only the spindle cell component. Major diagnostic pitfall is the presence of exuberant granulation tissue particularly on frozen section, fine needle aspiration (FNA) cytology, and incisional biopsy [16, 33]. Incisional biopsy material of our case also contained only spindle cells but clinical history revealed previous diagnosis of SCC and RT treatment as crucial data. Besides, tumor may imitate a true fibrosarcoma clearly or may have many blood vessels misleading to an angiosarcoma [18]. We also observed fibrosarcoma-like areas and the tumor was rich in blood vessels; but the presence of malignant squamous cells and history of the patient led us to the correct diagnosis. Additionally, high-proliferative index with ki-67 (MIB1) staining supported malignant stromal component rather than benign granulation tissue. 

In summary, we present here a rare case of SpCC arising from the tongue. Based on the limited number of studies in literature, its exact pathogenesis, clinical behavior, and long-term prognosis have not been well understood yet. Due to the increased conservative approaches in treatment and hence probable usage of radiotherapeutic modalities [34, 35], SpCC would likely be the diagnosis which surgical pathologist may confront more frequently in the future. Despite its challenging character, clinical history and presence of squamous cell component or determination of epithelial nature of spindle cells would guide the pathologist to the diagnosis. Finally, it should be remembered that pure sarcomas of the head and neck are extremely rare [5] and therefore spindle cell carcinoma should be always considered during evaluation of the polypoid lesions of this region containing spindle cells.
